# Impact of Sleeve Gastrectomy on Body Weight and Food Intake Regulation in Diet-Induced Obese Mice

**DOI:** 10.3390/cimb46110749

**Published:** 2024-11-07

**Authors:** Sandra Lucinei Balbo, Gabriela Moreira Soares, Joseane Morari, Antonio Machado Felisberto, Jean Franciesco Vettorazzi, Gabriela Alves Bronczek, Maria Lúcia Bonfleur, Everardo Magalhães Carneiro, Antonio Carlos Boschero, Lício Augusto Velloso

**Affiliations:** 1 Laboratory of Endocrine Physiology and Metabolism, Biological Sciences and Health Center, Western Parana State University, Cascavel 85819210, PR, Brazil; jrfelisberto@hotmail.com (A.M.F.J.); mlbonfleur@hotmail.com (M.L.B.); 2 Obesity and Comorbidities Research Center (OCRC), Department of Structural and Functional Biology, Institute of Biology, University of Campinas (UNICAMP), Campinas 13083864, SP, Brazil; moreirasoaresg@gmail.com (G.M.S.); morari@unicamp.br (J.M.); gabrielabronczek@gmail.com (G.A.B.); emc@unicamp.br (E.M.C.); boschero@unicamp.br (A.C.B.); lavellos@unicamp.br (L.A.V.); 3 Laboratory of Medical Sciences, Latin-American Institute of Life and Natural Sciences, Federal University of Latin-American Integration (UNILA), Foz do Iguassu 85867970, PR, Brazil

**Keywords:** obesity, bariatric surgery, food intake, hypothalamus, FGF15/19 pathway, foraging-like behavior

## Abstract

The epidemic of obesity has increased worldwide and is associated with comorbidities such as diabetes and cardiovascular disease. In this context, strategies that modulate body weight and improve glycemic metabolism have increased, and bariatric surgeries such as Sleeve Gastrectomy (SG) have been highlighted in obesity treatment. However, the mechanism by which SG reduces body weight and improves glycemic control remains unknown. Thus, in this study, we aimed to evaluate food intake and the expression of hypothalamic genes involved with the regulation of this process in diet-induced obese mice submitted to SG. For this, we used C57BL/6 mice submitted to a 10-week high-fat diet protocol and submitted to SG. Food intake, fed and fasted glycemia, as well as hypothalamic anorexigenic and orexigenic gene expression were evaluated 4 weeks after the surgical procedure. First, we observed that SG reduces body weight (44.19 ± 0.47 HFD, 43.51 ± 0.71 HFD-SHAM, and 38.22 ± 1.31 HFD-SG), fasting glycemia (115.0 ± 4.60 HFD, 122.4 ± 3.48 HFD-SHAM, and 93.43 ± 4.67 HFD-SG), insulinemia (1.77 ± 0.15 HFD, 1.92 ± 0.27 HFD-SHAM, and 0.93 ± 0.05 HFD-SG), and leptinemia (5.86 ± 1.38 HFD, 6.44 ± 1.51 HFD-SHAM, and 1.43 ± 0.35 HFD-SG) in obese mice. Additionally, SG reduces food (5.15 ± 0.18 HFD, 5.49 ± 0.32, HFD-SHAM, and 3.28 ± 0.26 HFD-SG) and total (16.88 ± 0.88 HFD, 17.05 ± 0.42, HFD-SHAM, and 14.30 ± 0.73 HFD-SG) calorie intake without alterations in anorexigenic and orexigenic gene expression. In conclusion, these data indicate that SG improves obesity-associated alterations at least in part by a reduction in food intake. This effect is not associated with the canonical food intake pathway in the hypothalamus, indicating the involvement of non-canonical pathways in this process.

## 1. Introduction

Obesity is a chronic multifactorial disease in which an accumulated excess of body fat leads to negative effects on health. The pathogenesis of obesity not only involves physiological factors, such as regulation of calorie utilization, appetite, and physical inactivity, but it also involves complex interactions with the availability of healthcare systems, the role of socio-economic status, and underlying hereditary and environmental factors [1]. The epidemic of obesity has reached elevated numbers worldwide, and it results in many diseases, such as type 2 diabetes mellitus (T2D), cardiovascular disease, chronic kidney disease, and cancer. In addition, obesity might lead to reduced quality of life, unemployment, lower productivity, and social disadvantages, which impact a patient’s quality of life [2]. This condition is associated with a chronic state of inflammation including peripheral tissues and the hypothalamus, a central area of the nervous system that regulates food intake [3,4].

Leptin and insulin are the main anorexigenic hormones acting via leptin and insulin receptors expressed in the central nervous system (CNS), especially in the hypothalamus [5,6,7]. These two hormones suppress the activity of orexigenic neurons NPY/AgRP, while stimulating anorexigenic POMC/CART neurons [8,9], thus controlling food intake. Resistance to the central actions of leptin or insulin is linked to increases in obesity and diabetes [10].

In this context, bariatric surgery has emerged as a potent tool in the prevention and treatment of obesity and T2D, offering significant and sustainable weight-loss outcomes. In addition, bariatric surgery also improves obesity-related comorbidities and significantly enhances patients’ quality of life [11,12]. This surgical approach includes various procedures that alter the anatomy of the gastrointestinal tract, leading to reduced food intake and nutrient absorption [13]. Nowadays, the most common bariatric surgery procedures are Roux-en-Y gastric bypass (RYGB) and Sleeve Gastrectomy (SG) [14,15]. Both surgeries reduce body weight and improve insulin signaling, as well as glucose homeostasis. In male Sprague-Dawley rats, RYGB increases mRNA levels of orexigenic genes AgRP and NPY, with no alterations in anorexigenic genes CART and POMC. Moreover, RYGB downregulates dopaminergic transmission markers [16]. However, the mechanism by which SG reduces body weight is not completely understood.

While the mechanism by which SG regulates body weight in this model remains unknown, this study aimed to evaluate food intake, as well as the expression of orexigenic and anorexigenic hypothalamic genes in diet-induced obese mice submitted to SG.

## 2. Materials and Methods

### 2.1. Animals

4-week-old male C57BL/6 mice obtained from the animal facility of the University of Campinas were maintained on a 12 h light/dark cycle in a temperature-controlled facility with free access to food and water. The Ethics Committee at the University of Campinas (License Number: 5242-1/2019) approved all experimental procedures involving mice, which were conducted in accordance with the last revision of the National Institutes of Health (NIH) guide for the care and use of laboratory animals.

### 2.2. Obesity Induction

Mice received a 45 kcal% saturated high-fat diet (HFD) (Prag Soluções; Jaú, SP, Brazil) for 10 weeks. Then, mice were randomly divided into three body-weight-matched groups prior to surgery: (1) high-fat diet group (HFD), (2) HFD submitted to sham operation (HFD-SHAM), and (3) HFD submitted to Sleeve Gastrectomy (HFD-SG).

### 2.3. Sleeve Gastrectomy and Sham Operations

Sleeve Gastrectomy (SG) and sham operations were performed under anesthesia as previously described [17]. After 12 h of fasting, mice were anesthetized with 1% isoflurane (BioChimico, Itatiaia, Brazil) with nasotracheal intubation (1 L/min O2). For the sham group, an incision was made in the epigastric midline of the abdomen; then, the stomach and abdominal cavity were exposed, and the small intestine was massaged using a sterile scalpel handle. Before suturing, a dose of 20 mg/kg of Enrofloxacin (Chemitril^®^, Chemitec^®^, São Paulo, SP, Brazil) and 5 mg/kg of Tramadol (Vitalis^®^, Bogotá, Colômbia) were administered to the abdominal cavity. The laparotomy was closed with a continuous suture with 6-0 polypropylene thread, as well as the skin. For SG, an incision was made in the epigastric midline of the abdomen, and the stomach was exposed. The incision was performed from the angle of His, and 80% of the volume of the stomach was removed, including complete resection of the gastric fundus, forming a gastric tube that connected the esophagus to the duodenum. Before suturing, a dose of 20 mg/kg Enrofloxacin (Chemitril^®^, Chemitec^®^, São Paulo, SP, Brazil) and Tramadol (Vitalis^®^, Bogotá, Colômbia) 5 mg/kg were administered to the abdominal cavity. The laparotomy was closed with a continuous suture with 6-0 polypropylene thread, as well as the skin. Mice received 20 mg/kg of Enrofloxacin (Chemitril^®^, Chemitec^®^, São Paulo, SP, Brazil) for 7 days after surgery, and 2 mg/kg of Meloxicam (Eurofarma^®^, São Paulo, SP, Brazil) plus 5 mg/kg of Tramadol (Vitalis^®^, Bogotá, Colômbia) for 2 days. Mice were kept on a liquid diet for 5 days after surgery. On day 6, mice were given doughy HFD, and they were switched back to solid HFD on day 12.

### 2.4. Glycemia, Insulin, and Leptin Evaluation

Four weeks after the postoperative recovery period, mice were weighed and glycemia was verified by a glucometer (Accu-chek^®^, Roche, Basileia, Switzerland) in fed and 12 h fasted states. Mice were euthanized by decapitation after isoflurane inhalation; the hypothalamus was removed for gene expression analysis, and plasma was collected for insulin and leptin measurement by ELISA kits (Mouse insulin, Catalog #10-1247-1, Mercodia, Sylveniusgatan, Sweden).

### 2.5. Food Intake

At the 3rd week after the postoperative recovery period, mice were maintained individually in home cages for 24 h of adaptation. After that, food consumption was measured during 3 consecutive days and was calculated by the difference between the food weight at 7 p.m. vs. 7 a.m. Food intake was then determined as the mean food consumption of this period [18].

### 2.6. mRNA Extraction and Real-Time Quantitative PCR (qRT-PCR)

The total RNA content of the hypothalamus was extracted using TRIzol reagent (Life Technologies, Gaithersburg, MD, USA), following phenol–chloroform RNA extraction, according to the manufacturer’s recommendations. RNA concentration was measured by a Nanodrop (Nanodrop Thermo Scientific, Wilmington, DE, USA). cDNA was prepared using 2 µg of total RNA and a high-capacity cDNA reverse transcription kit (Applied Biosystems, Foster City, CA, USA). A LuminoCt qPCR read mix (Sigma-Aldrich, Burlington, MA, USA) was used in the PCR reactions. Quantification was performed using a 7500 Fast Real-time PCR System (Applied Biosystems, Foster City, CA, USA). The relative expression of mRNAs was determined after normalization with the housekeeping gene *Gapdh* (Applied Biosystems, Foster City, CA, USA) using the 2^−ΔΔCt^ method. qRT-PCR target assays (IDT DNA Technologies, Ann Arbor, MI, USA) are shown in Table 1.

### 2.7. StatisticalAnalysis

Data are presented as the mean ± standard error of the mean (SEM). To evaluate data normality, we applied a Shapiro–Wilk test. When normal, we used One-Way ANOVA with an unpaired Tukey’s post-hoc test; otherwise, Kruskal–Wallis with an unpaired Dunn’s post-hoc test was adopted. The difference between groups was considered statistically significant if *p* ≤ 0.05.

## 3. Results

### 3.1. SG Reduces Body Weight, Fasting Glycemia, Insulinemia, and Leptinemia in Diet-Induced Obese Mice

Four weeks after SG, HFD-SG mice presented a reduction in body weight (38.22 ± 1.31) when compared to HFD and HFD-SHAM (44.19 ± 0.47 and 43.51 ± 0.71, respectively) (Table 1). HFD-SG mice also presented reduced fasting glycemia (93.43 ± 4.67), insulinemia (0.93 ± 0.05), and leptinemia (1.43 ± 0.35) when compared to HFD (115.0 ± 4.60, 1.77 ± 0.15, 5.86 ± 1.38, respectively) and HFD-SHAM (122.4 ± 3.48, 1.92 ± 0.27, 6.44 ± 1.51, respectively) (Table 2).

### 3.2. SG Reduces Food Intake with No Alterations in Hypothalamic Anorexigenic and Orexigenic Genes in Diet-Induced Obese Mice

**Figure 1:**
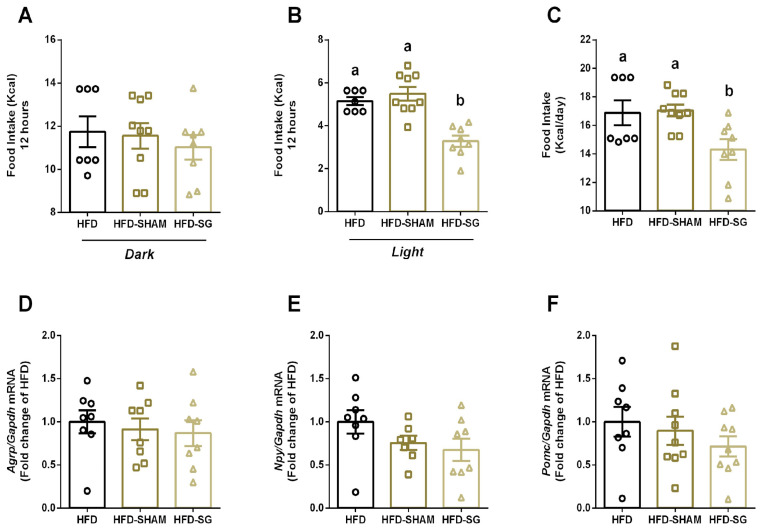
Sleeve gastrectomy reduces food intake with no alterations in hypothalamic neuropeptides in diet-induced obese mice. Food intake in Kcal for the 12 h dark period (**A**), the 12 h light period (**B**), and the total 24 h period (**C**) at the end of the experimental period. Real-time PCR assay of *Agrp* (**D**), *Npy* (**E**), and *Pomc* (**F**) mRNA levels in the hypothalamus from HFD, HFD-SHAM, and HFD-SG mice four weeks after the postoperative recovery period. The relative expression of mRNAs was determined after normalization with *Gapdh* using the 2^−ΔΔCt^ method. Data are the mean ± SEM (n = 7–9). Different letters indicate statistical differences between groups, *p* ≤ 0.05 (One-Way ANOVA with Tukey’s post-hoc test or Kruskal–Wallis with Dunn’s post-hoc test).

Once bariatric surgery reduced body weight, we aimed to evaluate food intake and regulation in mice. No differences were observed in food intake during the dark period between HFD, HFD-SHAM, and HFD-SG (11.74 ± 0.71; 11.56 ± 0.59; 11.02 ± 0.57, respectively) (Figure 1A). However, HFD-SG mice presented reduced food intake in the light period (3.28 ± 0.26) (Figure 1B), as well as reduced total calorie intake (14.30 ± 0.73) (Figure 1C) when compared to both HFD (5.15 ± 0.18; 16.88 ± 0.88) and HFD-SHAM mice (5.49 ± 0.32; 17.05 ± 0.42). Expression of orexigenic and anorexigenic genes in the hypothalamus was similar in all groups. (Figure 1D–F).

## 4. Discussion

In the present study, we verified that SG reduces insulin and leptin levels, as well as body weight. This effect is associated with reduced dark/light-cycle-dependent food intake, with no alterations in hypothalamic genes that regulate food intake. Thus, understanding the molecular mechanisms by which SG reduces body weight and improves glucose homeostasis contributing to improved quality of life is important in order to find new targets for obesity treatment.

Here, we observed that SG reduces food and Kcal intake during the light cycle (Figure 1B). It is known that rodents display different feeding behaviors than humans due to light/dark cycles, being more active in the night period [19]. However, when mice are exposed to a high-fat diet, they consume more food in their less active period (light period). This effect is associated with alterations in the expression of circadian rhythm genes in the liver and hypothalamus [20]. Furthermore, in middle-to-older-aged adult rats, the timing of food intake is associated with obesity development [21]. Also, in a rat model of nocturnal activity, shifting food intake to a natural period (dark cycle), which is the active phase, prevents obesity development [22]. Interestingly, our results showed that SG reduces food intake during the light period in obese mice, thus improving metabolism.

The hypothalamus plays a central role in food intake by the expression of orexigenic and anorexigenic genes [4]. However, no differences were observed in the expression of these genes in mice submitted to SG (Figure 1D–F). In contrast, RYGB surgery alters orexigenic gene expression with no differences observed in anorexigenic genes [16]. Thereby, SG modulates body weight, at least in part, by an orexigenic/anorexigenic-independent manner.

Recent studies have elucidated the effects of leptin and insulin interaction actions in the body. For instance, leptin resistance leads to the inhibition of insulin signaling, whereas insulin resistance alters leptin signaling in a hypothalamic cell line [23]. On the other hand, it is known that insulin potentiates the phosphorylation of STAT3 induced by leptin. STAT3 is a transcriptional factor critical to a major signaling pathway generating the anti-obesity effects of leptin [24]. In this sense, leptin and insulin may act synergistically, reducing body weight and food intake. Our data show that SG surgery reduces leptin and insulin levels (Table 1) as well as improves insulin sensitivity [17], which may be associated with reduced food intake.

Recently, it was demonstrated that SG reduces body weight and improves glucose homeostasis by the FGF15/19 pathway [17]. FGF15/19 is an intestinal hormone secreted from ileal enterocytes in response to a meal [25], and it has been shown that FGF15/19 levels increase in humans and mice submitted to bariatric surgery [26,27]. FGF19 plays a well-established role in bile acid metabolism; however, it also regulates energy and glucose homeostasis in rodents [28,29,30,31]. In obese mice, SG improves insulin sensitivity along with reduced beta cell insulin secretion. Also, SG reduces gene expression of inflammatory and ER stress markers in pancreatic beta cells [17]. This altered FGF15/19 pathway improves glucose homeostasis by reducing beta cell overload induced by a high-fat diet (HFD), which modulates pancreatic alpha cells, reducing glucagon secretion. This mechanism can contribute to avoiding the development of type 2 diabetes [17]. All these findings were observed 4 weeks after SG. However, other studies have demonstrated that 7 weeks after SG, body weight reduction still remains as well as an improvement in glucose homeostasis [32]. Moreover, diet-induced obese male Sprague-Dawley rats submitted to SG also present a reduction in body weight and food intake up until 8 weeks after the surgery [33].

FGF15/19 not only regulates body weight and peripheral glucose homeostasis, but it also acts centrally. The intracerebroventricular (ICV) effects of FGF19 in models of metabolic dysfunction have been studied in obese mice and rats [34,35], and it was observed that FGF19 acts on the hypothalamus, reducing food intake and body weight gain, and improving glucose tolerance and insulin resistance. Marcelin and colleagues also observed that central administration of FGF19 repressed AgRP/NPY neuron activation, improving glucose metabolism in obese mice orchestrated by FGF15/19-induced ERK1/2 signaling [34]. Corroborating the data, ICV administration of PD173074, which is a selective FGF receptor inhibitor, increases food intake and decreases glucose tolerance in rats [35]. These data suggest a physiological central role for the FGF15/19 signaling pathway.

Foraging for food precedes food consumption, and it is an important component of the overall metabolic program regulating feeding. Foraging is governed by neuronal circuits from the central nervous system [36,37,38]. Nevertheless, how this mechanism is influenced by diet and/or hormonal signals is still not well understood. Huang and colleagues have demonstrated that FGF19 suppresses foraging-like behaviors [39], suggesting that the intestinal hormone FGF15/19 signals a satiating state to the brain, thereby suppressing foraging-like behaviors.

Besides the effects of FGF15/19, other hormones such as oxytocin have also been explored in the SG context. A recent study has demonstrated that 12 months after SG, young men and women with severe obesity present reduced a body mass index (BMI) as well as lean and fat mass. The reduction in lean mass after the surgery was positively associated with reduced oxytocin levels. However, the mechanism by which SG modulates lean mass and the contribution of oxytocin in this context remain unknown [40].

The mechanism underlying reduced food intake by SG still needs to be clarified, but some hypotheses can be proposed. Bariatric surgery increases the release of many endocrine factors, including FGF15/19. This hormone has hypothalamic actions, altering feeding behavior and suppressing foraging-like behaviors. This effect suggests the involvement of a non-canonical pathway controlling food intake in the hypothalamus after bariatric surgery. However, further studies still have to be performed to investigate the role of FGF15/19 in controlling food intake and foraging-like behaviors in diet-induced obese mice.

## 5. Conclusions

Despite the promising findings obtained in this study, we only evaluated gene expression and hormone levels. We believe that further studies should validate the outcomes observed here at multiple levels, including protein expression and neurophysiological changes. Regardless of our limitations, our findings provide important insights into the contributions of SG surgery to control food intake since we observed that SG reduces food intake without altering the canonical regulation pathway in the hypothalamus. These findings may stimulate the scientific community to try and explore new pathways involved with the control of food intake and foraging-like behavior, thus shedding light on the development of non-surgical treatments in this field hereafter.

## Figures and Tables

**Table 1:** Primer sequences for real-time qPCR assays.

Gene	Assay	Ref Seq
*Agrp*	Mm.PT.58.31030782.g	NM_007427(2)
*Npy*	Mm.PT.58.29444574	NM_023456(1)
*Pomc*	Mm.PT.58.29397398	NM_008895(1)
*Gapdh*	Mm99999915_g1	NM_008084.2

*Agrp*: agouti-related neuropeptide; *Npy*: neuropeptide Y; *Pomc*: proopiomelanocortin; *Gapdh*: glyceraldehyde-3-phosphate dehydrogenase.

**Table 2:** Metabolic parameters of high-fat diet (HFD), HFD submitted to sham operation (HFD-SHAM), and HFD submitted to Sleeve Gastrectomy (HFD-SG) mice. Final body weight (n = 8–9), fasting glycemia (n = 7), fasting insulinemia (n = 7), and fasting leptinemia (n = 9) four weeks after the postoperative recovery period. Data are the mean  ±  SEM. Different letters indicate significant differences between groups (One-Way ANOVA with Tukey’s post-hoc test or Kruskal–Wallis with Dunn’s post-hoc test, *p*  ≤  0.05).

	HFD	HFD-SHAM	HFD-SG
Body weight (g)	44.19 ± 0.47 ^a^	43.51 ± 0.71 ^a^	38.22 ± 1.31 ^b^
Fasting glycemia (mg/dL)	115.0 ± 4.60 ^a^	122.4 ± 3.48 ^a^	93.43 ± 4.67 ^b^
Fasting insulinemia (ng/mL)	1.77 ± 0.15 ^a^	1.92 ± 0.27 ^a^	0.93 ± 0.05 ^b^
Fasting leptinemia (ng/mL)	5.86 ± 1.38 ^a^	6.44 ± 1.51 ^a^	1.43 ± 0.35 ^b^

## Data Availability

Data are contained within the article.

## References

[1] Blüher M. (2019). Obesity: Global epidemiology and pathogenesis. Nat. Rev. Endocrinol..

[2] Lin X., Li H. (2021). Obesity: Epidemiology, Pathophysiology, and Therapeutics. Front. Endocrinol..

[3] Sonnefeld L., Rohmann N., Geisler C., Laudes M. (2023). Is human obesity an inflammatory disease of the hypothalamus?. Eur. J. Endocrinol..

[4] Timper K., Brüning J.C. (2017). Hypothalamic circuits regulating appetite and energy homeostasis: Pathways to obesity. Dis. Models Mech..

[5] Baskin D.G., Breininger J.F., Schwartz M.W. (1999). Leptin receptor mRNA identifies a subpopulation of neuropeptide Y neurons activated by fasting in rat hypothalamus. Diabetes.

[6] Cheung C.C., Clifton D.K., Steiner R.A. (1997). Proopiomelanocortin Neurons Are Direct Targets for Leptin in the Hypothalamus. Endocrinology.

[7] Baskin D.G., Wilcox B.J., Figlewicz D.P., Dorsa D.M. (1988). Insulin and insulin-like growth factors in the CNS. Trends Neurosci..

[8] Morton G.J., Cummings D.E., Baskin D.G., Barsh G.S., Schwartz M.W. (2006). Central nervous system control of food intake and body weight. Nature.

[9] Schwartz M.W., Woods S.C., Porte D., Seeley R.J., Baskin D.G. (2000). Central nervous system control of food intake. Nature.

[10] Thon M., Hosoi T., Ozawa K. (2016). Possible Integrative Actions of Leptin and Insulin Signaling in the Hypothalamus Targeting Energy Homeostasis. Front. Endocrinol..

[11] Roth A.E., Thornley C.J., Blackstone R.P. (2020). Outcomes in Bariatric and Metabolic Surgery: An Updated 5-Year Review. Curr. Obes. Rep..

[12] Ji Y., Lee H., Kaura S., Yip J., Sun H., Guan L., Han W., Ding Y. (2021). Effect of Bariatric Surgery on Metabolic Diseases and Underlying Mechanisms. Biomolecules.

[13] Aderinto N., Olatunji G., Kokori E., Olaniyi P., Isarinade T., Yusuf I.A. (2023). Recent advances in bariatric surgery: A narrative review of weight loss procedures. Ann. Med. Surg..

[14] Buchwald H. (2014). The evolution of metabolic/bariatric surgery. Obes. Surg..

[15] Eisenberg D., Shikora S.A., Aarts E., Aminian A., Angrisani L., Cohen R.V., De Luca M., Faria S.L., Goodpaster K.P.S., Haddad A. (2022). 2022 American Society for Metabolic and Bariatric Surgery (ASMBS) and International Federation for the Surgery of Obesity and Metabolic Disorders (IFSO): Indications for Metabolic and Bariatric Surgery. Surg. Obes. Relat. Dis..

[16] Barkholt P., Pedersen P.J., Hay-Schmidt A., Jelsing J., Hansen H.H., Vrang N. (2016). Alterations in hypothalamic gene expression following Roux-en-Y gastric bypass. Mol. Metab..

[17] Soares G.M., Balbo S.L., Bronczek G.A., Vettorazzi J.F., Marmentini C., Zangerolamo L., Velloso L.A., Carneiro E.M. (2024). Vertical sleeve gastrectomy improves glucose-insulin homeostasis by enhancing β-cell function and survival via FGF15/19. Am. J. Physiol. Endocrinol. Metab..

[18] Soares G.M., Zangerolamo L., Costa-Júnior J.M., Vettorazzi J.F., Carneiro E.M., Saad S.T., Boschero A.C., Barbosa-Sampaio H.C. (2019). Whole-Body ARHGAP21-Deficiency Improves Energetic Homeostasis in Lean and Obese Mice. Front. Endocrinol..

[19] Desmet L., Thijs T., Mas R., Verbeke K., Depoortere I. (2021). Time-Restricted Feeding in Mice Prevents the Disruption of the Peripheral Circadian Clocks and Its Metabolic Impact during Chronic Jetlag. Nutrients.

[20] Kohsaka A., Laposky A.D., Ramsey K.M., Estrada C., Joshu C., Kobayashi Y., Turek F.W., Bass J. (2007). High-Fat Diet Disrupts Behavioral and Molecular Circadian Rhythms in Mice. Cell Metab..

[21] Xiao Q., Garaulet M., Scheer F.A.J.L. (2019). Meal timing and obesity: Interactions with macronutrient intake and chronotype. Int. J. Obes..

[22] Salgado-Delgado R., Angeles-Castellanos M., Saderi N., Buijs R.M., Escobar C. (2010). Food Intake during the Normal Activity Phase Prevents Obesity and Circadian Desynchrony in a Rat Model of Night Work. Endocrinology.

[23] Nazarians-Armavil A., Menchella J.A., Belsham D.D. (2013). Cellular Insulin Resistance Disrupts Leptin-Mediated Control of Neuronal Signaling and Transcription. Mol. Endocrinol..

[24] Thon M., Hosoi T., Ozawa K. (2016). Insulin enhanced leptin-induced STAT3 signaling by inducing GRP78. Sci. Rep..

[25] Guthrie G., Vonderohe C., Burrin D. (2022). Fibroblast growth factor 15/19 expression, regulation, and function: An overview. Mol. Cell. Endocrinol..

[26] Nemati R., Lu J., Dokpuang D., Booth M., Plank L.D., Murphy R. (2018). Increased Bile Acids and FGF19 After Sleeve Gastrectomy and Roux-en-Y Gastric Bypass Correlate with Improvement in Type 2 Diabetes in a Randomized Trial. Obes. Surg..

[27] Bozadjieva-Kramer N., Shin J.H., Shao Y., Gutierrez-Aguilar R., Li Z., Heppner K.M., Chiang S., Vargo S.G., Granger K., Sandoval D.A. (2021). Intestinal-derived FGF15 protects against deleterious effects of vertical sleeve gastrectomy in mice. Nat. Commun..

[28] Inagaki T., Choi M., Moschetta A., Peng L., Cummins C.L., Mcdonald J.G., Luo G., Jones S.A., Goodwin B., Richardson J.A. (2005). Fibroblast growth factor 15 functions as an enterohepatic signal to regulate bile acid homeostasis. Cell Metab..

[29] Zhou M., Luo J., Chen M., Yang H., Learned R.M., Depaoli A.M., Tian H., Ling L. (2017). Mouse species-specific control of hepatocarcinogenesis and metabolism by FGF19/FGF15. J. Hepatol..

[30] Matthew P.J., Boney-Montoya J., Choi M., He T., Sunny N.E., Satapati S., Suino-Powell K., Xu H.E., Gerard R.D., Finck B.N. (2011). FGF15/19 Regulates Hepatic Glucose Metabolism by Inhibiting the CREB-PGC-1α Pathway. Cell Metab..

[31] Kir S., Beddow S.A., Samuel V.T., Miller P., Previs S.F., Suino-Powell K., Xu H.E., Shulman G.I., Kliewer S.A., Mangelsdorf D.J. (2011). FGF19 as a Postprandial, Insulin-Independent Activator of Hepatic Protein and Glycogen Synthesis. Science.

[32] Ahn C.H., Choi E.H., Lee H., Lee W., Kim J.I., Cho Y.M. (2021). Vertical sleeve gastrectomy induces distinctive transcriptomic responses in liver, fat and muscle. Sci. Rep..

[33] Stefanidis A., Lee C.M.C., Greaves E., Montgomery M.K., Arnold M., Newn S., Budin A.J., Lemus M.B., Foldi C.J., Burton P.R. (2023). Mechanisms underlying the efficacy of a rodent model of vertical sleeve gastrectomy—A focus on energy expenditure. Mol. Metab..

[34] Marcelin G., Jo Y.H., Li X., Schwartz G.J., Zhang Y., Dun N.J., Lyu R.M., Blouet C., Chang J.K., Chua S. (2013). Central action of FGF19 reduces hypothalamic AGRP/NPY neuron activity and improves glucose metabolism. Mol. Metab..

[35] Ryan K.K., Kohli R., Gutierrez-Aguilar R., Gaitonde S.G., Woods S.C., Seeley R.J. (2013). Fibroblast growth factor-19 action in the brain reduces food intake and body weight and improves glucose tolerance in male rats. Endocrinology.

[36] Aponte Y., Atasoy D., Sternson S.M. (2011). AGRP neurons are sufficient to orchestrate feeding behavior rapidly and without training. Nat. Neurosci..

[37] Krashes M.J., Koda S., Ye C., Rogan S.C., Adams A.C., Cusher D.S., Maratos-Flier E., Roth B.L., Lowell B.B. (2011). Rapid, reversible activation of AgRP neurons drives feeding behavior in mice. J. Clin. Investig..

[38] Luquet S., Perez F.A., Hnasko T.S., Palmiter R.D. (2005). NPY/AgRP neurons are essential for feeding in adult mice but can be ablated in neonates. Science.

[39] Huang A., Maier M.T., Vagena E., Xu A.W. (2023). Modulation of foraging-like behaviors by cholesterol-FGF19 axis. Cell Biosci..

[40] Becetti I., Singhal V., Nimmala S., Lee H., Lawson E.A., Bredella M.A., Misra M. (2023). Serum Oxytocin Levels Decrease 12 Months Following Sleeve Gastrectomy and Are Associated with Decreases in Lean Mass. Int. J. Mol. Sci..

